# A computer-aided detection system in the everyday setting of diagnostic, screening, and surveillance colonoscopy: an international, randomized trial

**DOI:** 10.1055/a-2328-2844

**Published:** 2024-06-27

**Authors:** Michiel H. J. Maas, Timo Rath, Cristiano Spada, Elsa Soons, Nauzer Forbes, Sergey Kashin, Paola Cesaro, Axel Eickhoff, Geoffroy Vanbiervliet, Daniele Salvi, Paul J. Belletrutti, Peter D. Siersema, for the Discovery study team

**Affiliations:** 1 Gastroenterology and Hepatology, Radboud University Medical Center, Nijmegen, the Netherlands; 2 Department of Medicine I, Division of Gastroenterology, Universitätsklinikum Erlangen, Erlangen, Germany; 3 Department of Gastroenterology and Endoscopy, Fondazione Poliambulanza Istituto Ospedaliero, Brescia, Italy; 4 Università Cattolica del Sacro Cuore, Fondazione Policlinico Universitario A. Gemelli IRCCS, Rome, Italy; 5 Department of Medicine, University of Calgary, Calgary, Canada; 6 Department of Endoscopy, Yaroslavl Regional Cancer Hospital, Yaroslavl, Russia; 7 Gastroenterology, Diabetology, Infectiology, Klinikum Hanau, Hanau, Germany; 8 Pôle digestif, Hôpital L’Archet, Nice, France; 9 ErasmusMC – University Medical Center, Rotterdam, the Netherlands

## Abstract

**Background** Computer-aided detection (CADe) has been developed to improve detection during colonoscopy. After initial reports of high efficacy, there has been an increasing recognition of variability in the effectiveness of CADe systems. The aim of this study was to evaluate a CADe system in a varied colonoscopy population.

**Methods** A multicenter, randomized trial was conducted at seven hospitals (both university and non-university) in Europe and Canada. Participants referred for diagnostic, non-immunochemical fecal occult blood test (iFOBT) screening, or surveillance colonoscopy were randomized (1:1) to undergo CADe-assisted or conventional colonoscopy by experienced endoscopists. Participants with insufficient bowel preparation were excluded from the analysis. The primary outcome was adenoma detection rate (ADR). Secondary outcomes included adenomas per colonoscopy (APC) and sessile serrated lesions (SSLs) per colonoscopy.

**Results** 581 participants were enrolled, of whom 497 were included in the final analysis: 250 in the CADe arm and 247 in the conventional colonoscopy arm. The indication was surveillance in 202/497 colonoscopies (40.6 %), diagnostic in 199/497 (40.0 %), and non-iFOBT screening in 96/497 (19.3 %). Overall, ADR (38.4 % vs. 37.7 %; *P* = 0.43) and APC (0.66 vs. 0.66; *P* = 0.97) were similar between CADe and conventional colonoscopy. SSLs per colonoscopy was increased (0.30 vs. 0.19; *P* = 0.049) in the CADe arm vs. the conventional colonoscopy arm.

**Conclusions** In this study conducted by experienced endoscopists, CADe did not result in a statistically significant increase in ADR. However, the ADR of our control group substantially surpassed our sample size assumptions, increasing the risk of an underpowered trial.

## Introduction

Colonoscopy is considered the gold standard for the detection and removal of premalignant colorectal lesions. Despite its effectiveness, a notable number of lesions are still missed during colonoscopy 1 , increasing the risk of post-colonoscopy colorectal cancer (CRC) 2 . This risk is inversely correlated with the adenoma detection rate (ADR), which is widely considered the main quality parameter in colonoscopy 3 4 .

Recently, artificial intelligence systems, such as computer-aided detection (CADe) systems, have emerged to assist in the detection of colorectal polyps during colonoscopy. A recent systematic review of 21 randomized controlled trials (RCTs) assessing CADe vs. conventional colonoscopy in over 18 000 patients demonstrated an approximate 24 % relative increase in ADR due to CADe 5 . Despite this significant benefit in overall ADR, there were no significant differences between CADe and conventional colonoscopy in the detection of advanced adenomas or sessile serrated lesions (SSLs) 5 , raising concerns regarding the efficacy of CADe in these lesions with a higher risk of CRC. Furthermore, despite reports of high efficacy in RCTs, there has been an increasing recognition of the variability in the performance of CADe systems across different colonoscopy indications and pragmatic trials 6 7 .

The aim of this study was to compare ADR and other quality indicators in CADe-assisted colonoscopy vs. conventional colonoscopy in patients with diagnostic, non-immunochemical fecal occult blood test (iFOBT) screening, or surveillance indications for colonoscopy.

## Methods

### Study design and participants

This multicenter RCT, involving seven hospitals in Canada (n = 1), France (n = 1), Germany (n = 2), Italy (n = 1), The Netherlands (n = 1), and Russia (n = 1), was conducted by 14 endoscopists. Eligible participants, aged 18 years or older, were scheduled for non-iFOBT screening, surveillance, or diagnostic (excluding iFOBT-positive referrals) colonoscopy. Exclusion criteria included known colorectal tumors or polyps upon referral, referral for therapeutic procedures, inadequately corrected coagulation disorder, inadequately continued use of anticoagulation medication, American Society of Anesthesiologists score of ≥ 3, or known or suspected inflammatory bowel disease. Participants with insufficient bowel preparation (Boston Bowel Preparation Scale score < 6), active colitis, polyposis syndrome, colonic stricture, or obstructing CRC impeding complete colonoscopy were excluded from the final analysis. Participants in the Yaroslavl, Russia, study site enrolled after 24 February 2022, were excluded from the final analysis following a directive from the Dutch Federation of University Hospitals, mandating the temporary suspension of all collaborations with Russian study sites.

The study received approval from independent institutional review boards at each site, adhered to the Declaration of Helsinki, and followed applicable Good Clinical Practice guidelines. Data verification and monitoring complied with national and local guidelines where appropriate. The study was reported in accordance with CONSORT-AI guidelines for RCTs, and all participants provided written informed consent. All authors had access to the study data and reviewed and approved the final manuscript.

### Randomization

Participants were randomized after eligibility was assessed and informed consent was obtained. Participants were randomized in a 1:1 ratio to either CADe-assisted colonoscopy or conventional colonoscopy. Randomization employed varying block sizes of 4, 6, and 8. Stratification for randomization was based on whether the subject was undergoing an index colonoscopy, defined as the first lifetime colonoscopy of a participant. Randomization was performed on-site, within 24 hours before the scheduled colonoscopy, by a central, cloud-based randomization service (CastorEDC; Ciwit B.V., Amsterdam, The Netherlands). Endoscopists, participants, and the data analyst were not blinded to the study allocation.

### Artificial intelligence system

**Fig. 1:**
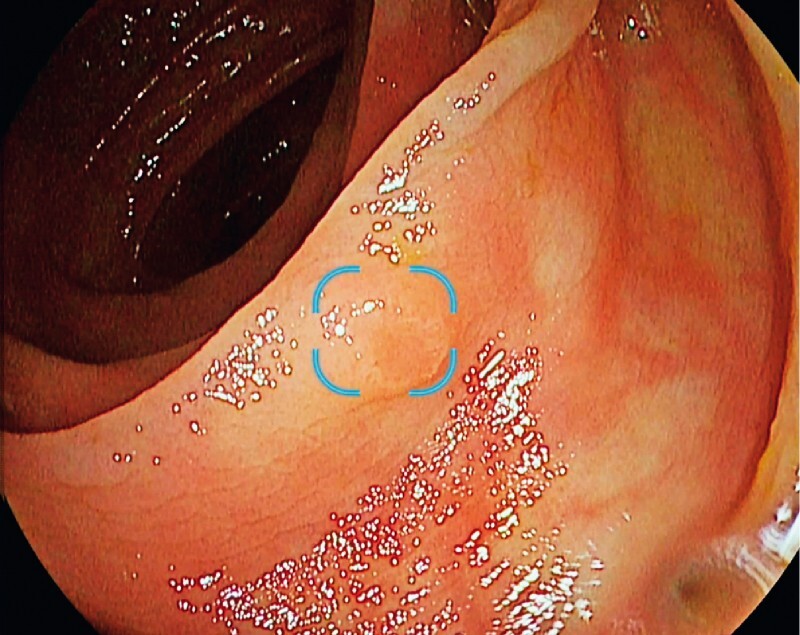
The computer-aided detection system generates an overlay during real-time colonoscopy, with a blue bounding box to highlight a lesion.

The CADe DISCOVERY system (PENTAX Medical, Tokyo, Japan) used for the CADe-assisted colonoscopies is a real-time computing device that acquires the video output from the processor during colonoscopy. The CADe system uses a deep neural network to generate a bounding box around a suspected polyp as an output on the monitor screen in real time ( Fig. 1 ). The system is used as an auxiliary device and aims to improve the detection rate by highlighting potential lesions. Final assessment of the highlighted region was the responsibility of the endoscopist. The endoscopist could choose to be acoustically notified of detections. During the study, CADe software versions 1.0.3.1 and 1.0.4 were used.

### Study investigators

All endoscopists underwent training in CADe-assisted colonoscopy, completing a minimum of five CADe procedures to confirm their familiarity with the system. The study was conducted at sites with an annual performance exceeding 5000 colonoscopies. Participation was limited to experienced endoscopists to mitigate potential improvements in ADR due to training throughout the study. Endoscopists were eligible if they had independently performed over 500 colonoscopies, reflecting procedural experience rather than a specific minimum ADR. This approach aimed to approach the real-world variability in ADR among endoscopists. Notably, all endoscopists had completed over 2000 independent colonoscopies prior to the start of the study.

### Study procedures

In the CADe arm, the device was switched on at the beginning of the CADe colonoscopy, and the use of CADe was mandatory during the withdrawal phase. Endoscopists were advised to primarily use the CADe monitor; however, the use of a dual monitor setup, with separate displays for the conventional image and the CADe overlay, was not explicitly prohibited.

Each study site used local bowel preparation protocols and sedation administration. All participants randomized to conventional colonoscopy underwent colonoscopy as per standard of care. During the study, conventional PENTAX high-definition colonoscopes were used for both arms. To ensure adequate bowel inspection, all participating endoscopists were instructed to aim for a minimum withdrawal time of 6 minutes (excluding time spent on polypectomies or other interventions) following societal guidelines 3 . In addition, an upper withdrawal time of 10 minutes was recommended to reflect everyday procedural scheduling and reduce observation-related bias.

All lesions were collected for histopathologic examination in separate containers for each polyp. Diminutive (1–5 mm) polyps located in the rectum and considered to be hyperplastic by the performing endoscopist could be left in place according to endoscopists’ individual judgment and standard of care. Experienced pathologists, who were blinded to the endoscopic diagnosis, determined the histopathologic diagnosis according to the Vienna classification 8 .

### Study outcomes

The primary outcome was ADR, calculated as the proportion of colonoscopies with at least one histologically confirmed detected adenoma. Additionally, ADR was evaluated across various variables, including colonoscopy indication and in a per-endoscopist analysis. Secondary outcomes were mean number of adenomas per colonoscopy (APC; total number of histologically confirmed adenomas divided by the total number of colonoscopies), polyp detection rate (proportion of colonoscopies with at least one histologically confirmed detected polyp), sessile serrated lesions (SSLs) per colonoscopy (total number of histologically confirmed SSLs divided by the total number of colonoscopies), and SSL detection rate (proportion of colonoscopies with at least one histologically confirmed detected SSL). Other secondary outcomes included withdrawal time without interventions and the number of false positives during CADe-assisted colonoscopy (defined as a non-neoplastic, non-hyperplastic area highlighted by CADe for > 3 consecutive seconds). The reasons for false positives were reported and were calculated as the number of CADe colonoscopies with at least one subcategory of the reason for false positives.

### Sample size calculation

This study was powered to detect a significant difference in ADR. The sample size calculation was performed using G*Power, version 3.1.9.7 (Heinrich-Heine-Universität, Düsseldorf, Germany). During the design of the study protocol, only one RCT had been published regarding the effect of CADe on ADR, reporting an increase in ADR from 20.3 % to 29.1 % 9 . With this limited prior data, we set the baseline ADR at 18 % for conventional colonoscopy, expecting a 50 % relative increase with CADe to 27 %. Furthermore, we expected no decrease in detection given the working mechanism of CADe, as it is used as an auxiliary device to conventional colonoscopy, enhancing the displayed image output without directly interfering with colonoscope handling. Consequently, we assumed a one-directional effect and used a one-sided test for sample size calculations. Using a one-sided *Z* test for independent proportions (5 % alpha, 80 % power), the sample size was 532 participants. To account for a dropout rate of 5 %, the final sample size was set at 560, evenly distributed between the two study arms (280 each).

### Statistical analysis

Analyses of the primary and secondary outcomes followed a modified intention-to-treat approach, excluding participants with an inadequate Boston Bowel Preparation Scale score or for whom a quality colonoscopy could not be performed. Analysis of the primary outcome was performed using the chi-squared test, dividing the two-sided *P* value by two to calculate the one-sided *P* value. Statistical analyses were performed using SPSS 27 (IBM Corp., Armonk, New York, USA) or R Studio 4.1.3 (R Foundation for Statistical Computing, Vienna, Austria).

Continuous variables were presented as means (SD) or medians (interquartile range [IQR]), and categorical data as numbers/percentages. Differences between study arms for secondary outcomes were assessed using *t* tests, Mann–Whitney *U* tests, or chi-squared tests, as appropriate. Two-sided *P* values were reported for the secondary outcomes. Wilson Score Method was used to calculate 95 %CIs where applicable. A logistic regression model evaluated ADR. Predetermined potential confounding factors, including sex, age, body mass index, smoking status, reason for colonoscopy, and study site, were excluded from the model following study protocol, as these variables appeared evenly distributed across study arms. Sensitivity analysis compared APC and SSLs per colonoscopy using Poisson regression. Post hoc analysis explored the effect of CADe among low, medium, and high detectors, categorizing endoscopists based on ADR tertiles. Additionally, to address our relatively high dropout rate, a post hoc analysis of the primary outcome was conducted on an intention-to-treat cohort. Statistical significance was set at *P* < 0.05, unless otherwise specified.

## Results

**Fig. 2:**
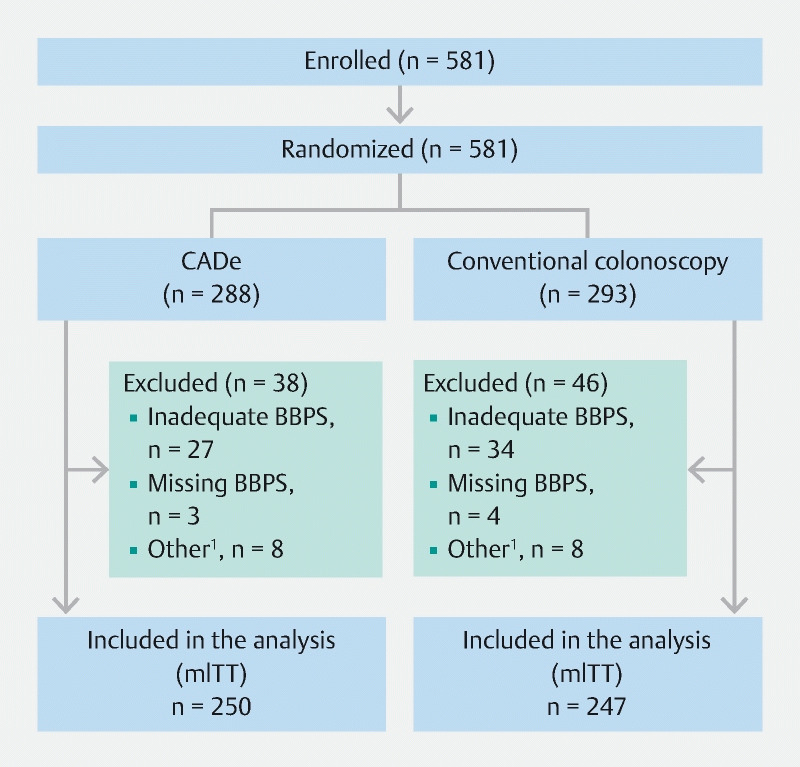
Study flow chart. CADe, computer-aided detection; BBPS, Boston Bowel Preparation Scale; mITT, modified intention-to-treat. ^1^ Other exclusions: Russian site enrollment after 24 February 2022 (n = 12), new polyposis diagnosis (n = 2), American Society of Anesthesiologists score of 3 (n = 1), or new diagnosis of inflammatory bowel disease (n = 1).

The study was performed from 9 March 2021 to 6 February 2023. The relatively long inclusion period was partly related to the COVID-19 pandemic in the initial period of the study. A total of 581 participants were enrolled and randomized (1:1) to either CADe (n = 288) or conventional colonoscopy (n = 293). A total of 84 participants were excluded, leaving 497 participants in the final analysis (modified intention-to-treat): 250 participants in the CADe arm and 247 in the conventional colonoscopy arm ( Fig. 2 ). While the dropout rate of our modified intention-to-treat analysis (14.5 %; 497/581) exceeded our expected dropout rate, we were unable to replace these participants due to institutional review board guidelines. Baseline characteristics were similar between the study arms ( Table 1 ). No missing values were observed for the calculation of the primary and secondary outcomes. All procedures in the CADe arm were performed with the CADe modality activated.

**Table 1:** Baseline characteristics in the modified intention-to-treat population.

	CADe (n = 250)	Conventional colonoscopy (n = 247)
Age, median (IQR), years	61.0 (52–69)	61.0 (52–69)
Sex, n (%)		
Female	141 (56.4)	136 (55.1)
Male	109 (43.6)	111 (44.9)
Colonoscopy indication, n (%)		
Screening (non-iFOBT)	50 (20.0)	46 (18.6)
Surveillance	98 (39.2)	104 (42.1)
Diagnostic ^1^	102 (40.8)	97 (39.3)
Index colonoscopy, n (%)	112 (44.8)	108 (43.7)
Smoking, n (%)	26 (10.4)	32 (13.0)
Family history of CRC, n (%)	60 (24.0)	45 (18.2)
BMI ^2^ , kg/m ^2^	25.5 (23.1–28.3)	25.0 (22.5–28.8)
BBPS score, n (%)		
6	61 (24.4)	65 (26.3)
7	27 (10.8)	27 (10.9)
8	29 (11.6)	34 (13.8)
9	133 (53.2)	121 (49.0)

CADe, computer-aided detection; IQR, interquartile range; iFOBT, immunochemical fecal occult blood test; CRC, colorectal cancer; BMI, body mass index; BBPS, Boston Bowel Preparation Scale.

^1^ Diagnostic indications did not include iFOBT-positive referrals. ^2^ Weight was missing for one patient.

### Overall findings

ADR was similar between the CADe arm and the conventional colonoscopy arm (38.4 % vs. 37.7 %, *P* = 0.43; total colonoscopies with at least one adenoma, 96 vs. 93) ( Table 2 ). Logistic regression analysis calculated an odds ratio (OR) of 1.032 (95 %CI 0.719–1.483) for CADe relative to conventional colonoscopy ( Table 3 ). Similarly, APC was comparable between the CADe arm and the conventional colonoscopy arm (0.66 vs. 0.66, *P* = 0.97; total detected adenomas 165 vs. 163). While polyp detection rate was numerically increased in the CADe arm compared with the conventional colonoscopy arm, the difference was not significant (55.2 % vs. 51.4 %, *P* = 0.40; total colonoscopies with at least one polyp, 138 vs. 127). Furthermore, SSLs per colonoscopy was significantly higher in the CADe arm compared with the conventional colonoscopy arm (0.30 vs. 0.19, *P* = 0.049; total detected SSLs, 76 vs. 46) and SSL detection rate was increased in the CADe arm compared with the conventional colonoscopy arm (18.4 % vs. 12.1 %; *P* = 0.053, total colonoscopies with at least one SSL, 46 vs. 30, respectively). Median withdrawal time was similar between study arms (withdrawal time without interventions [IQR] 9.2 [8.0–11.0] vs. 9.0 [8.0–11.0] minutes, *P* = 0.05, for CADe and conventional colonoscopy, respectively).

**Table 2:** Primary and secondary outcomes in the modified intention-to-treat population.

	CADe (n = 250)	Conventional colonoscopy (n = 247)	Difference (treatment – control) [95 %CI] ^1^	*P* value
Adenoma detection rate ^2^ , % (n)	38.4 (96)	37.7 (93)	0.7 [–7.8 to 9.3]	0.43
Adenoma per colonoscopy, n (n/N)	0.66 (165 /250)	0.66 (163 /247)	0.00 [–0.19 to 0.19]	0.97
Polyp detection rate, % (n)	55.2 (138)	51.4 (127)	3.8 [–5.0 to 12.5]	0.40
SSLs per colonoscopy, n (n/N)	0.30 (76 /250)	0.19 (46 /247)	0.11 [0.00 to 0.24]	0.049
SSL detection rate, % (n)	18.4 (46)	12.1 (30)	6.3 [–0.04 to 12.5]	0.05
Mean polyps per colonoscopy, n (n/N)	1.20 (299 /250)	1.09 (270 /247)	0.11 [–0.15 to 0.36]	0.52
Withdrawal time without interventions, median (IQR), minutes	9.2 (8.0–11.0)	9.0 (8.0–11.0)	0.2	0.05
Total procedure time, median (IQR), minutes	20.0 (15.0–27.6)	20.0 (15.0–24.7)	0.0	0.43

CADe, computer-aided detection; SSL, sessile serrated lesion; IQR, interquartile range.

^1^ 95 %CI calculated using Wilson score interval for proportions. ^2^ Statistical analysis of the primary outcome was performed using a one-sided approach to the chi-squared test. Other *P* values represent two-sided analyses.

**Table 3:** Additional analysis of primary and secondary outcomes in the modified intention-to-treat population.

	CADe (n = 250)	Conventional colonoscopy (n = 247)	OR/effect ratio CADe to conventional colonoscopy [95 %CI]	*P* value
Adenoma detection rate ^1^ , %, (n)	38.4 (96)	37.7 (93)	1.032 [0.719 to 1.483]	0.86
Mean number of adenomas per colonoscopy ^2^ , n (n/N)	0.66 (165 /250)	0.66 (163 /247)	1.006 [0.811 to 1.249]	0.96
SSLs per colonoscopy ^3^ , n (n/N)	0.30 (76 /250)	0.19 (46 /247)	1.632 [1.132 to 2.354]	0.01
Mean number of polyps per colonoscopy ^3^ , n (n/N	1.20 (299 /250)	1.09 (270 /247)	1.098 [0.863 to 1.397]	0.45

CADe, computer-aided detection; OR, odds ratio; SSL, sessile serrated lesion.

^1^ OR from logistic binary regression with treatment arm and adenoma detection rate. ^2^ Effect ratio from Poisson regression with log-link function. ^3^ Effect ratio from Poisson regression with negative binomial function.

When stratified by colonoscopy indication the results were similar. For diagnostic colonoscopies (n = 199), ADR was increased by 5.5 percentage points in the CADe arm compared with the conventional colonoscopy arm (33.3 % vs. 27.8 %, *P* = 0.40; total colonoscopies with at least one adenoma, 34 vs. 27), and for SSLs per colonoscopy the increase was 0.09 in the CADe arm compared with the conventional colonoscopy arm (0.25 vs. 0.16, *P* = 0.15; total detected SSLs, 26 vs. 16). For surveillance colonoscopies (n = 202), ADR in the CADe arm was equal to that in the conventional colonoscopy arm (43.9 % vs. 43.9 %, *P* = 0.93; total colonoscopies with at least one adenoma, 43 vs. 45), and for SSLs per colonoscopy the increase was 0.15 in the CADe arm compared with the conventional colonoscopy arm (0.36 vs. 0.21, *P* = 0.41; total detected SSLs, 35 vs. 22). For non-iFOBT screening colonoscopies (n = 96), ADR was decreased by 7.7 percentage points in the CADe arm compared with the conventional colonoscopy arm (38.0 % vs. 45.7 %, *P* = 0.45; total colonoscopies with at least one adenoma, 19 vs. 21), and for SSLs per colonoscopy the increase was 0.13 in the CADe arm compared with the conventional colonoscopy arm (0.30 vs. 0.17, *P* = 0.23; total detected SSLs, 15 vs. 8). Additional outcomes are reported in Table 2 and Table 3 (see also **Tables 1s–4 s** in the online-only Supplementary material).

During the withdrawal phase of the CADe-assisted colonoscopy, the median number of false positives was 2.0 (IQR 0.0–5.0; mean 4.1 [SD 6.1]). Colonic haustral folds were the most frequently reported reason for false positives during CADe-assisted colonoscopy (40.8 %) ( **Table 5 s** ).

## Discussion

In this multicenter RCT involving experienced endoscopists from both university and non-university hospitals, the use of CADe did not significantly increase ADR or APC in diagnostic, non-iFOBT screening, or surveillance colonoscopies. However, despite the non-significant increase in adenoma detection, use of the CADe system resulted in an absolute increase of 0.11 (relative increase 58 %) of SSLs per colonoscopy compared with conventional colonoscopy.

Our study did not find a significant increase in ADR or APC with CADe, which contrasts with previously published Western RCTs, as well as a recent meta-analysis of 21 RCTs including over 18 000 patients, which reported an absolute ADR difference of 8.1 % (44.0 % vs. 35.9 %) with CADe compared with conventional colonoscopy 5 10 11 12 13 14 15 . Moreover, one of the earliest RCTs on CADe, by Repici et al., reported an absolute increase of 14.4 % in ADR among expert endoscopists 16 . However, a recent non-university, single-center study by Karsenti et al., with over 2000 participants, reported an ADR of 37.5 % with CADe, which was similar to that found in our study. In their conventional colonoscopy arm, the baseline ADR was 33.7 %, resulting in an absolute difference of only 3.8 % with the use of CADe. Their study had a similar proportion of diagnostic and screening colonoscopies as reported in our study 15 .

Our non-significant increase in adenoma detection is consistent with recently published controlled and real-world studies. An RCT conducted in a screening and surveillance population at four US community hospitals reported a non-significant increase in APC from 0.67 to 0.73, comparable to our study 17 . A pragmatic implementation trial performed by Ladabaum et al., which employed CADe during all colonoscopies without specific instructions, reported no significant differences in detection rates 6 . Notably, their baseline ADR was comparable to that in our study. Moreover, their study did not identify differences in the efficacy of CADe between low detectors and high detectors. Similarly, Levy et al. integrated CADe into all colonoscopies at their high-volume tertiary referral center. Their study also did not find a significant increase in adenoma detection compared with a retrospective cohort 7 . While our study employed an RCT design, the study period extended over nearly 2 years, which may have resulted in a reduced level of constant scrutiny and observation. This intermittent exposure to the CADe device could have reduced the Hawthorne effect while using CADe, which is a potential source of bias in controlled studies 18 . The extended duration of our study, combined with the variable colonoscopy indications, may reflect a more real-world clinical setting compared with previous positive RCTs on CADe that had substantially shorter study durations 9 12 16 .

On the other hand, our non-significant results may be attributed to the relatively high baseline ADR in the conventional colonoscopy arm, approaching 38 %. As a result, this may have limited the potential beneficial effect of CADe, as endoscopists with a higher ADR might derive less benefit from CADe compared with their peers with a lower ADR 5 15 19 . We also observed this trend in our post hoc analysis comparing low, medium, and high detectors based on their baseline ADR, although no statistically significant difference was observed ( **Table 6 s, Fig. 1 s** ); however, owing to the post hoc nature of this analysis, findings should be interpreted with caution. Moreover, the median withdrawal time in our study significantly exceeded the recommended 6 minutes and approached the upper-end target of 10 minutes 3 20 . Our relatively long withdrawal times may have contributed to the high baseline ADR of our conventional colonoscopy arm, as each additional minute has been shown to be associated with an increase in ADR; however, this effect seems to diminish after 10 minutes 21 22 . Finally, while our study is the first RCT evaluating this CADe system, we are cautious about attributing the non-significant results to the potential lack of stand-alone efficacy of the system. While most CADe RCTs have reported an increase in ADR with CADe use, some RCTs did not find an increase 23 24 , despite previous positive results using the same CADe system 10 . This suggests that factors beyond the CADe system are important when interpreting these results. Nonetheless, our findings suggest a potential increase in detection, as indicated by the significant increase in SSLs per colonoscopy and a trend toward increased polyp detection rates. However, additional studies are required to provide a more comprehensive understanding of the performance of this CADe system.

The clinical relevance of our study is supported by the increased detection of SSLs, as reflected by the significant relative 58 % increase of SSLs per colonoscopy and a borderline significant but absolute increase of 6.3 % in the SSL detection rate in the CADe arm. Although our overall SSL detection rate of 12.1 % in the conventional colonoscopy arm might appear relatively high, it is comparable to previously published CADe studies with variable indications 6 12 23 . This relatively high SSL detection rate is not unexpected, given the increasing awareness and recognition of SSLs, as demonstrated by the steady increase in SSL detection rates since 2008 25 . Furthermore, in a retrospective analysis of the training and evaluation sets of this CADe system (unpublished results), we found that 11 % of the used lesions were diagnosed as SSLs. This relatively large proportion of SSLs in the training set may have contributed to our significant increase in the detection of these lesions, which are notably hard to detect. Our study is, to the best of our knowledge, the first to demonstrate a significant increase in the detection of SSLs with the use of CADe. These SSLs are nowadays recognized for their clinical importance in the CRC pathway, and the SSL detection rate is increasingly recognized as a potential quality parameter in colonoscopy 26 . Furthermore, a recent study has shown that endoscopists with an increased SSL detection rate have a lower risk of post-colonoscopy CRC, even when corrected for ADR 27 . However, we acknowledge that the detection of SSLs was a secondary outcome in our study.

The strengths of this study include the balanced distribution of participants across six countries, in both Europe and Canada, among both university and non-university hospitals. This approach reduced the potential risk of bias associated with endoscopists who conduct a substantial number of procedures and show significantly improved detection with CADe. In addition, the inclusion criteria reflect everyday colonoscopy populations by incorporating varied colonoscopy indications. Furthermore, distal attachments were not used. Finally, the validation of the CADe system was not performed on the included study populations of participating study sites, reducing the risk of potential overfitting, which is a well-known risk of artificial intelligence systems.

However, our study has some limitations. First, in hindsight, our assumptions for the sample size calculation were rather conservative. Initially, we assumed a baseline ADR of only 18 % in the conventional colonoscopy arm, influenced by limited data, notably a single Chinese CADe RCT 9 . Despite our initial assumptions proving to be underestimated, particularly with the inclusion of experienced endoscopists, our total calculated sample size did not significantly differ from early Western CADe studies 10 16 28 . Additionally, detecting a significant result with our modest difference in ADR would require a substantially larger sample size, as exemplified by a recent RCT with over 2000 participants, where use of CADe resulted in a borderline significant absolute increase in ADR of only 3.8 % ( *P* = 0.05) 15 . Nevertheless, we acknowledge the potential increased risk of a type II statistical error resulting from our sample size calculation. Second, pathology slides were not evaluated by a second, independent, expert pathologist. This could have introduced some bias in the diagnosis of SSLs in our study, considering that even expert pathologists have only a moderate interobserver agreement when diagnosing SSLs 29 . Nonetheless, this risk is likely to be limited because the pathologists in our study demonstrated proficiency in recognizing SSLs, as indicated by our relatively high detection rates of SSLs compared with previous CADe RCTs 9 10 11 16 . Third, the modified intention-to-treat analysis included 497 (85.5 %) of the 581 colonoscopies, reflecting a higher than anticipated exclusion rate due to insufficient or missing Boston Bowel Preparation Scale scores. This could be attributed to the varied colonoscopy indications and non-standardized bowel preparation. While our dropout rate was higher than expected, the subsequent potential risk of further underpowering in the study appears to be limited, as supported by the similar results of the intention-to-treat analysis ( **Table 7 s** ). To mitigate the risk of exclusion due to insufficient bowel preparation in future studies, randomizing after reaching the cecum could be considered. Fourth, although training recommended primarily using the CADe monitor in a single-monitor setup, a dual-monitor setup displaying both the conventional image and CADe output side by side was not prohibited, potentially influencing gaze patterns in the selected cases where such a setup was used 30 .

In conclusion, use of CADe by experienced endoscopists did not result in an increased ADR and APC in everyday diagnostic, non-iFOBT screening, and surveillance colonoscopies in our study. CADe increased the detection of SSLs, which are notoriously hard to detect and are increasingly recognized for their clinical relevance; however, SSLs per colonoscopy was not a primary outcome in our study.

## Supplementary Material

Supplementary material Zusatzmaterial
