# Supplementary material for: A computer-aided detection system in the everyday setting of diagnostic, screening, and surveillance colonoscopy: an international, randomized trial
Source: Endoscopy. 2024 Jun 27;56(11):843–50. doi: 10.1055/a-2328-2844 (PMC11524745; doi:10.1055/a-2328-2844)

Supplementary material

A computer-aided detection system in the everyday setting of diagnostic, screening, and surveillance colonoscopy: an international, randomized trial

M.H.J. Maas, T. Rath, C. Spada, E. Soons, N. Forbes, S. Kashin, P. Cesaro, A. Eickhoff, G. Vanbiervliet, D. Salvi, P.J. Belletrutti, P.D. Siersema; for the Discovery study team.

Supplementary material

Table 1s Primary and secondary outcomes per reason for colonoscopy in the modified intention-to-treat population

	Conventional colonoscopy (n=247)	CADe (n=250)	Difference (treatment – control)	P value
**Adenoma detection rate (ADR)**				
Overall*	93/247 = 37.7%	96/250 = 38.4%	0.7 [-7.8 – 9.3]	.432
Non-iFOBT screening (n=96)	21/46 = 45.7%	19/50 = 38.0%	-7.7 [-27.4 – 12.1]	.447
Surveillance (n=202)	45/104 = 43.3%	43/98 = 43.9%	0.6 [-13.1 – 14.3]	.931
Diagnostic (n=199)	27/97 = 27.8%	34/102 = 33.3%	5.5 [-7.3 – 18.3]	.400
**Adenoma per colonoscopy (APC)**				
Overall	163/247 = 0.66	165/250 = 0.66	0.00 [-0.19 – 0.19]	.971
Non-iFOBT screening (n=96)	37/46 = 0.80	47/50 = 0.94	0.14 [-0.42 – 0.69]	.748
Surveillance (n=202)	84/104 = 0.81	71/98 = 0.72	-0.09 [-0.40 – 0.23]	.699
Diagnostic (n=199)	42/97 = 0.43	48/102 = 0.47	0.04 [-0.18 – 0.25]	.459
**Polyp detection rate (PDR)**				
Overall	127/247 = 51.4%	138/250 = 55.2%	3.8 [-5.0 – 12.5]	.398
Non-iFOBT screening (n=96)	27/46 = 58.7%	30/50 = 60.0%	1.3 [-18.4 – 21.0]	.897
Surveillance (n=202)	58/104 = 55.8%	59/98 = 60.2%	4.4 [-9.2 – 18.4]	.523
Diagnostic (n=199)	42/97 = 43.3%	49/102 = 48.0%	4.7 [-9.1 – 18.6]	.502
**Sessile serrated lesions per colonoscopy (SSLPC)**				
Overall	46/247 = 0.19	76/250 = 0.30	0.11 [0.00 – 0.24]	.049

Supplementary material

Non-iFOBT screening (n=96)	8/46 = 0.17	15/50 = 0.30	0.13 [-0.13 – 0.38]	.227
Surveillance (n=202)	22/104 = 0.21	35/98 = 0.36	0.15 [-0.09 – 0.38]	.405
Diagnostic (n=199)	16/97 = 0.16	26/102 = 0.25	0.09 [-0.07 – 0.25]	.150
**Sessile serrated lesion detection rate (SDR)**				
Overall	30/247 = 12.1%	46/250 = 18.4%	6.3 [-0.04 – 12.5]	.053
Non-iFOBT screening (n=96)	5/46 = 10.9%	10/50 = 20.0%	9.1 [-5.1 – 23.4]	.218
Surveillance (n=202)	14/104 = 13.5%	17/98 = 17.3%	3.8 [-6.1 – 13.8]	.444
Diagnostic (n=199)	11/97 = 11.3%	19/102 = 18.6%	7.3 [-2.6 – 17.1]	.151
**Mean Polyps per colonoscopy (PPC)**				
Overall	270/247 = 1.09	299/250 = 1.20	0.11 [-0.15 – 0.36]	.515
Non-iFOBT screening (n=96)	59/46 = 1.28	80/50 = 1.60	0.32 [-0.38 – 1.02]	.608
Surveillance (n=202)	135/104 = 1.30	125/98 = 1.28	-0.02 [-0.45 – 0.41]	.886
Diagnostic (n=199)	75/97 = 0.77	94/102 = 0.92	0.15 [-0.16 – 0.46]	.358
**Withdrawal time without interventions (min)**	9.0 [8.0 – 11.0]	9.2 [8.0 – 11.0]	0.2	.052
**Total Procedure time (min)**	20.0 [15.0 – 24.7]	20.0 [15.0 – 27.6]	0.0	.430

*Statistical analysis of the primary outcome is performed using a one-sided approach to the Chi-square test. Other p-values represent two-sided analyses. Data is n/N(%), or median(IQR). CADe=computer-aided detection, CC=conventional colonoscopy, [95%CI] calculated using Wilson score interval for proportions.

Supplementary material

Table 2s Adenoma detection rate characteristics in the modified intention-to-treat population

	CC (n=247)	CADe (n=250)	Difference (treatment – control)	P value
**Localization**				
Caecum	15/247 = 6.1%	13/250 = 5.2%	-0.9 [-4.9 – 3.2]	.673
Ascending colon	33/247 = 13.4%	48/250 = 19.2%	5.8 [-0.6 – 12.3]	.078
Transverse colon	32/247 = 13.0%	32/250 = 12.8%	-0.2 [-6.0 – 5.7]	.959
Descending colon	21/247 = 8.5%	12/250 = 4.8%	-3.7 [-8.1 – 0.7]	.097
Sigmoid colon	25/247 = 10.1%	18/250 = 7.2%	-2.9 [-7.9 – 2.0]	.247
Rectum	8/247 = 3.2%	14/250 = 5.6%	2.4 [-1.2 – 6.0]	.201
Proximal colon	63/247 = 25.5%	72/250 = 28.8%	3.3 [-4.5 – 11.1]	.409
Distal colon	49/247 = 19.8%	41/250 = 16.4%	-3.4 [-10.2 – 3.3]	.320
**Size**				
≤5mm	67/247 = 27.1%	77/250 = 30.8%	3.7 [-4.3 - 11.6]	.367
6-9mm	38/247 = 15.4%	28/250 = 11.2%	-4.2 [-10.1 – 1.8]	.169
≥10mm	14/247 = 5.7%	14/250 = 5.6%	-0.1 [-0.4 – 4.0]	.974
**Morphology (Paris classification)**				
Pedunculated	11/247 = 4.5%	9/250 = 3.6%	-0.9 [-4.3 – 2.6]	.628
Sessile	72/247 = 29.2%	73/250 = 29.2%	0.0 [-7.9 – 8.0]	.990
Flat elevated	17/247 = 6.9%	29/250 = 11.6%	4.7 [-0.4 – 9.8]	.070
Flat lesion	5/247 = 2.0%	4/250 = 1.6%	-0.4 [-2.8 – 1.9]	.723

Supplementary material

Slightly depressed	0/247 = 0.0%	0/250 = 0.0%	-	-
Excavated	0/247 = 0.0%	0/250=0.0%	-	-

CC=conventional colonoscopy, CI=confidence interval, CADe=computer-aided detection. Data are n/N(%). [95% CI] are calculated using the Wilson score interval for proportions.

Supplementary material

Table 3s ADR per study site in the modified intention-to-treat population

Study site	CC (n=247)	CADe (n=250)	Difference (treatment – control)	P value
01 (n=112)	22/57 = 38.6%	25/55 = 45.5%	6.9 [-11.4 – 25.1]	.462
02 (n=85)	17/39 = 43.6%	15/46 = 32.6%	-11.0 [-31.6 – 9.7]	.298
03 (n=108)	18/54 = 33.3%	24/54 = 44.4%	11.1 [-7.2 – 29.4]	.236
04 (n=109)	21/54 = 38.9%	20/55 = 36.4%	-2,5 [-20.7 – 15.7]	.786
05 (n=23)	4/12 = 33.3%	4/11 = 36.4%	3.1 [-36.0 – 42.0]	.879
06 (n=38)	8/20 = 40.0%	4/18 = 22.2%	-17.8 [-46.6 – 11.0]	.239
07 (n=22)	3/11 = 27.3%	4/11 = 36.4%	9.1 [-29.6 – 47.8]	.647

CADe=computer-aided detection, CC=conventional colonoscopy, CI=confidence interval. Data are n/N(%). [95% CI] are calculated using the Wilson score interval for proportions.

Supplementary material

Table 4s ADR per endoscopist in the modified intention-to-treat population

Endoscopist	CC (n=247)	CADe (n=250)	Difference (treatment – control)	P value
001	17/30 = 56.7%	11/23 = 47.8%	-8.9 [-35.8 – 18.2]	.523
002	3/15 = 20.0%	8/17 = 47.1%	27.1 [-4.1 – 58.2]	.108
003	0/5 = 0.0%	5/10 = 50%	50.0 [19.0 – 81.0]	.053
004	2/7 = 28.6%	1/5 = 20.0%	-8.6 [-57.0 – 39.9]	.753
005	16/43 = 37.2%	21/43 = 48.8%	11.6 [-9.2 – 32.4]	.276
006	2/7 = 28.6%	2/6 = 33.3%	4.7 [-45.7 – 55.2]	.853
007	0/3 = 0.0%	1/3 = 33.3%	33.3 [-20.0 – 86.7]	.273
008	0/1 = 0.0%	0/2 = 0.0%	-	-
009	17/39 = 43.6%	15/46 = 32.6%	-11.0 [-31.6 – 9.7]	.298
010	8/28 = 28.6%	10/33 = 30.3%	1.7 [30.3 – 28.6]	.883
011	13/26 = 50.0%	10/22 = 45.5%	-4.5 [-32.9 – 23.8]	.753
012	4/12 = 33.3%	4/11 = 36.4%	3.1 [-36.0 – 42.0]	.879
013	3/11 = 27.3%	4/11 = 36.4%	9.1 [-29.6 – 47.8]	.647
014	8/20 = 40.0%	4/18 = 22.2%	-17.8 [-46.6 – 11.0]	.239

CADe=computer-aided detection, CC=conventional colonoscopy, CI=confidence interval. Data are n/N(%). [95% CI] are calculated using the Wilson score interval for proportions.

Supplementary material

Table 5s False positives in the modified intention-to-treat population

	**CADe (n=250)**
**False positives, median (IQR) [mean, ±SD]**	2.0 (0.0 – 5.0) [4.1 ± 6.1]
**Reason of false positive per colonoscopy, n (%)**	
Colonic fold	102/250 = 40.8%
Bubble	77/250 = 30.8%
Fecal material	82/250 = 32.8%
Ileocecal valve	27/250 = 10.8%
Suction artefact	16/250 = 6.4%
Other	27/250 = 10.8%

CADe=computer-aided detection. False positives were characterized as an unsuspected area highlighted by CADe for longer than 3 seconds, as assessed by the endoscopist.

Supplementary material

Table 6s Post-hoc analysis of ADR between CC and CADe-assisted colonoscopy according to endoscopist basal ADR

	CC (n=247)	CADe (n=250)	Difference (treatment – control)	P value
**Adenoma detection rate (ADR)**				
Lower-detector tertile	15/65 = 23.1% [14.5 – 34.6]	27/74 = 36.5% [26.4 – 47.9]	13.4 [-1.6 – 28.4]	.086
Medium-detector tertile	28/75 = 37.3% [27.3 – 48.6]	29/72 = 40.3% [29.7 – 51.8]	3.0 [-12.8 – 18.7]	.714
High-detector tertile	47/95 = 49.5% [39.6 – 59.4]	36/91 = 39.6% [30.1 – 49.8]	-9.9 [-24.1 – 4.3]	.174

Endoscopists were categorized in tertiles based on their ADR in the CC study arm. Endoscopists with <5 colonoscopies performed in the CC study arm were excluded from the initial calculation of tertiles. Subsequently, they were added to their corresponding tertile based on their ADR. Low-detectors were the endoscopists in the bottom tertile, medium-detectors in the middle tertile, and high-detectors were the top tertile. ADR=adenoma detection rate, CADe=computer-aided detection, CC=conventional colonoscopy.

Supplementary material

Table 7s Primary and secondary outcomes of the intention-to-treat population

	CC (n=290)	CADe (n=287)	Difference (treatment – control)	P value
**Adenoma detection rate (ADR)**				
Overall*	105/290 = 36.2%	106/287 = 36.9%	0.7 [-7.1 – 8.6]	.428
Non-iFOBT screening (n=107)	23/52 = 44.2%	21/55 = 38.2%	-6.0 [-24.7 – 12.6]	.525
Surveillance (n=234)	51/124 = 41.1%	47/110 = 42.7%	1.6 [-11.1 – 14.2]	.805
Diagnostic (n=236)	31/114 = 27.2%	38/122 = 31.1%	3.4 [-7.6 – 15.5]	.504
**Adenoma per colonoscopy (APC)**				
Overall	177/290 = 0.61	184/287 = 0.64	0.03 [-0.14 – 0.20]	.911
Non-iFOBT screening (n=107)	39/52 = 0.75	50/55 = 0.91	0.16 [-0.35 – 0.67]	.537
Surveillance (n=234)	91/124 = 0.73	81/110 = 0.74	0.01 [-0.29 – 0.29]	.980
Diagnostic (n=236)	47/114 = 0.41	53/122 = 0.43	0.02 [-0.17 – 0.22]	.557
**Polyp detection rate (PDR)**				
Overall	147/290 = 50.7%	158/287 = 55.1%	4.4 [-3.8 – 12.5]	.294
Non-iFOBT screening (n=107)	31/52 = 59.6%	33/55 = 60.0%	0.4 [-18.2 – 19.0]	.968
Surveillance (n=234)	67/124 = 54.0%	67/110 = 60.9%	6.9 [-5.8 – 19.5]	.289
Diagnostic (n=224)	49/114 = 43.0%	58/122 = 47.5%	4.3 [-8.1 – 17.3]	.482
**Sessile serrated lesions per colonoscopy (SSLPC)**				
Overall	56/290 = 0.19	85/287 = 0.30	0.11 [0.00 – 0.21]	.045
Non-iFOBT screening (n=107)	9/52 = 0.17	16/55 = 0.29	0.12 [-0.11 – 0.35]	.235

Supplementary material

Surveillance (n=234)	23/124 = 0.19	36/110 = 0.33	0.14 [-0.05 – 0.35]	.320
Diagnostic (n=236)	24/114 = 0.21	33/122 = 0.27	0.06 [-0.1 – 0.22]	.202
**Sessile serrated lesion detection rate (SDR)**				
Overall	36/290 = 12.4%	53/287 = 18.5%	6.1 [0.2 – 11.9]	.044
Non-iFOBT screening (n=107)	6/52 = 11.5%	11/55 = 20.0%	9.5 [-5.2 – 22.1]	.231
Surveillance (n=234)	15/124 = 12.1%	18/110 = 16.4%	4.3 [-4.7 – 13.3]	.349
Diagnostic (n=236)	15/114 = 13.2%	24/122 = 19.7%	6.5 [-2.9 – 15.9]	.178
**Mean Polyps per colonoscopy (PPC)**				
Overall	304/290 = 1.05	335/287 = 1.17	0.12 [-0.12 – 0.35]	.388
Non-iFOBT screening (n=107)	65/52 = 1.25	86/55 = 1.56	0.31 [-0.33 – 0.95]	.569
Surveillance (n=234)	148/124 = 1.19	139/110 = 1.26	0.07 [-0.32 – 0.46]	.708
Diagnostic (n=236)	91/114 = 0.80	110/122= 0.90	0.10 [-0.19 – 0.40]	.444
**Withdrawal time without interventions (min)**	9.0 (8.0 – 11.0)	9.0 (8.0 – 11.0)	0.0	.026
**Total Procedure time (min)**	19.0 (15.0 – 24.0)	20.0 (15.0 – 27.0)	1.0	.225

ITT population (n=577) consists of all randomized patients (n=581) after exclusion of new polyposis diagnosis (n=2), ASA score of 3 (n=1), or new IBD diagnosis (n=1). *Statistical analysis of the primary outcome is performed using a one-sided approach to the Chi-square test. Other p-values represent two-sided analyses. CADe=computer-aided detection, CC=conventional colonoscopy, CI=confidence interval. Data are n/N(%) or median (IQR). [95% CI] are calculated using the Wilson score interval for proportions.

Supplementary material

Fig. 1s

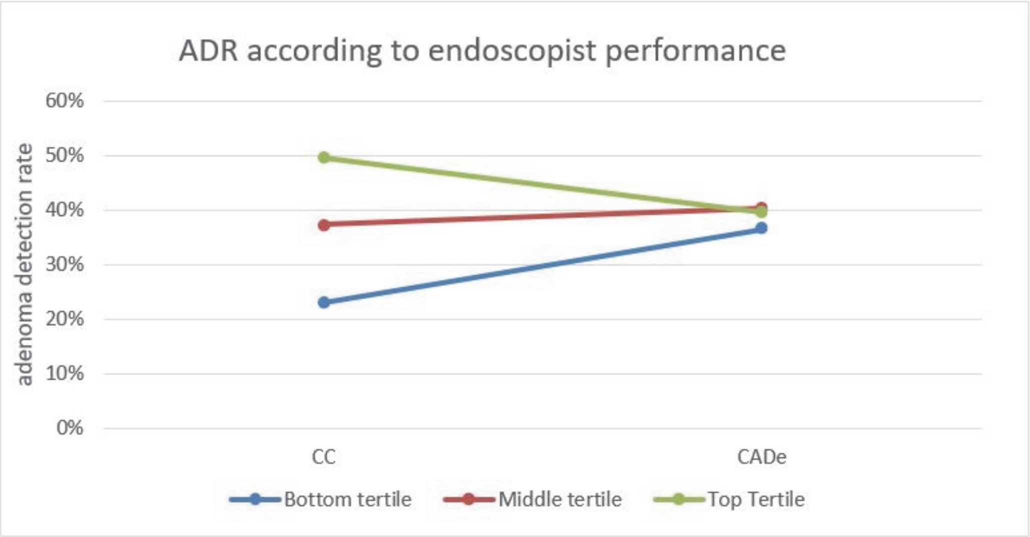

Supplement: Supplementary file 1 — Supplementary material [file 23852supmat_10-1055-a-2328-2844.pdf]
